# From Origin to the Present: Establishment, Mechanism, Evolutions and Biomedical Applications of the CRISPR/Cas-Based Macromolecular System in Brief

**DOI:** 10.3390/molecules30040947

**Published:** 2025-02-18

**Authors:** Zheng Yuan

**Affiliations:** Institute of Chinese Materia Medica, China Academy of Chinese Medical Sciences, Beijing 100022, China; zyuan@icmm.ac.cn

**Keywords:** gene editing, CRISPR/Cas macromolecular system, gene therapy, bioanalytical methods

## Abstract

Advancements in biological and medical science are intricately linked to the biological central dogma. In recent years, gene editing techniques, especially CRISPR/Cas systems, have emerged as powerful tools for modifying genetic information, supplementing the central dogma and holding significant promise for disease diagnosis and treatment. Extensive research has been conducted on the continuously evolving CRISPR/Cas systems, particularly in relation to challenging diseases, such as cancer and HIV infection. Consequently, the integration of CRISPR/Cas-based techniques with contemporary medical approaches and therapies is anticipated to greatly enhance healthcare outcomes for humans. This review begins with a brief overview of the discovery of the CRISPR/Cas system. Subsequently, using CRISPR/Cas9 as an example, a clear description of the classical molecular mechanism underlying the CRISPR/Cas system was given. Additionally, the development of the CRISPR/Cas system and its applications in gene therapy and high-sensitivity disease diagnosis were discussed. Furthermore, we address the prospects for clinical applications of CRISPR/Cas-based gene therapy, highlighting the ethical considerations associated with altering genetic information. This brief review aims to enhance understanding of the CRISPR/Cas macromolecular system and provide insight into the potential of genetic macromolecular drugs for therapeutic purposes.

## 1. Introduction

The central dogma of molecular biology, which describes the flow of genetic information from DNA to RNA to protein, has been widely accepted by scientists in the fields of physiology and medicine [1,2]. Over time, the central dogma has undergone refinement through the efforts of projects such as the Human Genome Project and the Human Epigenome Project [3,4]. And these advancements have led to the development and improvement of various disciplines including genetics, epigenetics, and bioinformatics. Furthermore, the applications of the central dogma have expanded to encompass numerous areas of medicine and pharmaceutical research, such as antimicrobial therapy, immunotherapy, gene therapy, and new drug discovery [5,6]. Therefore, the continuous refinement and application of the central dogma are crucial for healthcare and medical innovation.

According to the central dogma of molecular biology, the proteins responsible for phenotypic functions are primarily determined by the original DNA sequence of genes. Therefore, gene editing, which involves purposefully altering the gene sequence, enables the desired expression of functional proteins in cells, leading to a change in cellular phenotypic function. Notably, in the last two decades, significant advancements have been made with the discovery of the clustered regularly interspaced short palindromic repeats (CRISPR), the Cas9 protein, and the elucidation of Cas9–RNA–DNA interactions. This breakthrough has led to the development of a new gene editing technology based on the CRISPR/Cas system, which offers high efficiency and low resource consumption [7,8,9,10]. The CRISPR/Cas-based strategy has successfully enabled gene editing in various types of prokaryotic and eukaryotic cells, and has also facilitated disease biomarker detection [11]. As a result, it is anticipated that with ongoing development, the CRISPR/Cas-based system will be further integrated into modern medical therapies to address current challenging diseases. To summarize, the advancements of the CRISPR/Cas system in gene editing technologies hold great promise for expanding the scope of medical interventions and treatments. Moreover, the CRISPR/Cas system continues to undergo rapid development, with constant improvements in efficiency, specificity, and delivery methods. Concurrently, the field is witnessing an accelerating push towards clinical translation, moving CRISPR/Cas from a research tool to a potential therapeutic modality. Given this dynamic landscape, a timely review that captures the current advancements and prospects is essential to guide researchers and clinicians.

**Scheme 1:**
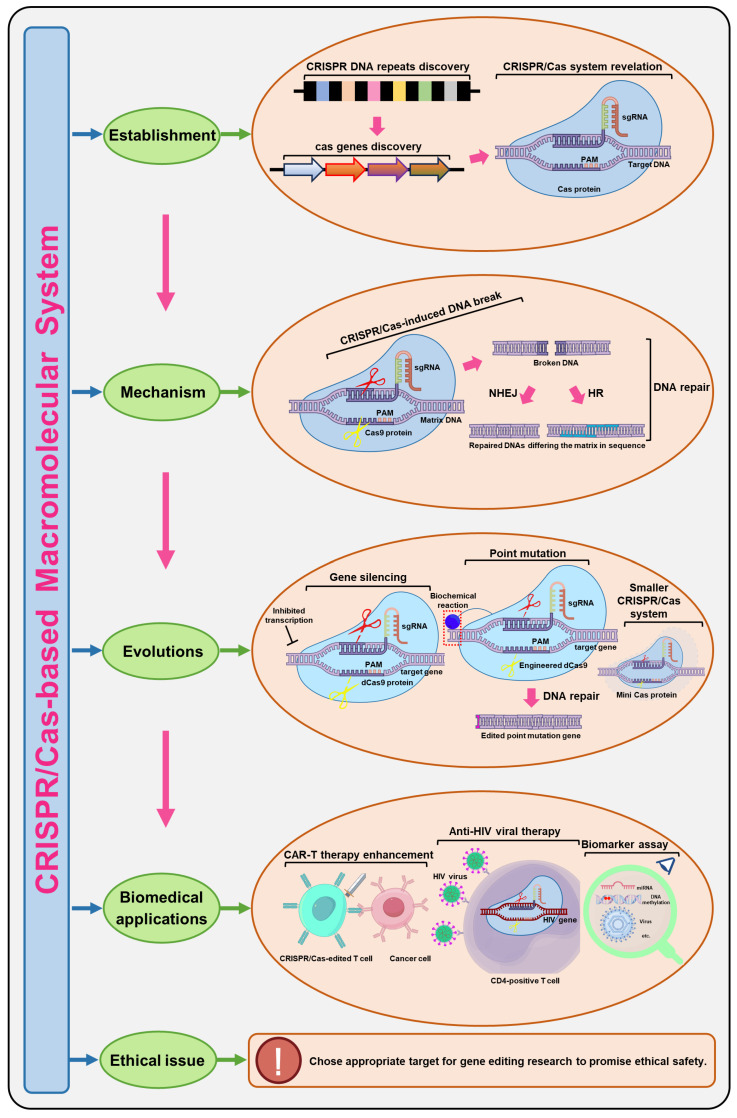
The brief organization and description associate with the CRISPR/Cas-based macromolecular system discussed in the review.

This review will initially focus on the historical background of the CRISPR/Cas system to elucidate its evolution as a recognized gene editing tool. Subsequently, the molecular mechanism underlying gene editing mediated by Cas9, which holds paramount importance within the Cas protein family, will be thoroughly elucidated. In addition to CRISPR/Cas9, the evolutions of CRISPR/Cas systems were introduced. Furthermore, the diverse applications of CRISPR/Cas in biomedical therapy and disease biomarker diagnosis will be introduced. Finally, beyond discussing the prospects, the review delves into the potential ethical issues intrinsic to CRISPR/Cas technology that distinguish it from previous medical technologies. The brief organization and description of the review were present in Scheme 1. This essay aims to give a comprehensive but brief introduction to the developing CRISPR/Cas-based techniques and induce ethical considerations arising from gene editing.

## 2. Construction History of the CRISPR/Cas-Based Gene Editing System

### 2.1. Discovery and Mystery of CRISPR

In 1987, Ishino et al. discovered a series of DNA repeats in the *Escherichia coli* (*E. coli*) genome consisting of highly homologous sequences with 29 base pairs [12]. These repeats were located in non-coding regions and did not attract significant research interest at that time. However, 15 years later, they were named clustered regularly interspaced short palindromic repeats (CRISPR) when CRISPR-associated (cas) genes were discovered [13]. The CRISPR phenomenon was also observed in other prokaryotic genomes such as Archaea [14]. Mojica et al. made a breakthrough by aligning and analyzing DNA sequences. They found that CRISPR spacers showed high homology with sequences from bacteriophages, which are viruses that infect prokaryotic cells like *E. coli* [15]. Based on this homology, Mojica et al. inferred a connection between prokaryotic CRISPR and its immunity against exogenous viral DNA.

### 2.2. Cas9 Protein and Associated Binding RNAs

Subsequently, the focus of CRISPR research shifted to cas genes, which encode a big family of Cas proteins [16,17]. Haft et al. extensively characterized four Cas proteins (Cas1, Cas2, Cas3, and Cas4) through bioinformatics analysis [16]. Of particular significance is the functional characterization of Cas9 protein in establishing the CRISPR/Cas gene editing system. Marraffini et al. reported the existence of small CRISPR RNAs (crRNAs) transcribed from CRISPR sequences and demonstrated crRNAs-dependent autoimmunity in *Staphylococcus epidermidis* against foreign DNA interference [18]. Garneau et al. demonstrated the indispensable role of Cas protein expression in *Streptococcus thermophilus* for conferring immunity against foreign plasmids or phages [19]. Additionally, Deltcheva et al. discovered the essential involvement of tracrRNA (trans-activating CRISPR RNA), formed in *Streptococcus pyogenes*, in protecting against prophage DNA invasion by interacting with the Cas9 protein [20]. Thus, unraveling the specific molecular mechanism underlying CRISPR-mediated self-protection against foreign DNA infections necessitates elucidating the intricate interplay among Cas9 protein, tracrRNA, crRNA, and exogenous DNA.

### 2.3. Revelation of the CRISPR/Cas System

Encouragingly, a significant advancement was made by Jinek et al. when they elucidated the intricate molecular mechanism underlying complex interactions. They discovered that a protein–RNA complex, consisting of Cas9 protein, tracrRNA and complementary crRNA, facilitates the double-stranded DNA break (DSB) of double-stranded DNA (dsDNA) through recognition of the protospacer adjacent motif (PAM) sequences and homologous sequences within the crRNA (the detailed process will be present in the next section) [21]. Additionally, Jinek et al. demonstrated that a single RNA molecule, formed by linking tracrRNA and crRNA, can also associate with Cas9 to perform the same function of mediating DSB [21]. Importantly, as DSB often induce DNA repair process that can modify genomic DNA sequences in vivo, they proposed that the CRISPR/Cas9 system holds immense potential as a tool for precisely altering gene sequences at targeted loci, namely gene editing [21]. Subsequently, Cong et al. reported successful application of the CRISPR/Cas system for multiple gene editing in eukaryotes at the cellular level [22]. Although numerous advancements and updates have been made in the CRISPR/Cas system research afterward, the gene editing capabilities of the CRISPR/Cas9 system were thoroughly explained and established up to this point.

## 3. Detail Process of CRISPR/Cas-Mediated Gene Editing

A successful and CRISPR/Cas-mediated gene editing involves two key steps: (1) the CRISPR/Cas system mediates a site-specific DSB in the genomic DNA, and (2) the cell activates the DNA damage repair (DDR) system to repair the DSB, resulting in a modified DNA sequence. In this section, we will discuss these two steps in detail, focusing on Cas9 protein-mediated gene editing.

### 3.1. Targeted DSB by the CRISPR/Cas System

Understanding the sequences of participated nucleic acid including crRNA, tracrRNA and target dsDNA, and the structure of Cas9 protein is crucial. The crRNA typically consists of 20–24 bases and encompasses a sequence complementary to one single-stranded DNA (ssDNA) of the target dsDNA at its 5′ segment, while its 3′ segment contains another sequence complementary to one single-stranded RNA (ssRNA) part of tracrRNA [23]. The tracrRNA is an elongated ssRNA with approximately 75–170 bases [24]. Apart from processing a complementary segment to the 3′ end of the crRNA, which forms a stem–loop structure, tracrRNA tend to form three additional stem–loop structures using its remaining bases [25]. Notably, by adhering to specific sequence and structural requirements, crRNA and tracrRNA can be fused together into a single-guide RNA (sgRNA) using a short RNA linker consisting of a few bases. In the case of the target dsDNA, in addition to having a complementary segment with crRNA, the adjacent downstream sequence of the non-complementary ssDNA must contain a brief sequence known as protospacer adjacent motif (PAM), which typically consists of 2–6 base pairs. For the Cas9 protein-based gene editing system, the PAM sequence is generally NGG, where G represents the guanine deoxybase, and N denotes any of the four DNA deoxybases (adenine: A, G, cytosine: C, and thymidine: T) [21]. The Cas9 protein itself is a sizable molecule weighing approximately 160 kDa and comprising 1100 to 1400 amino acid residues, contingent upon its biological origins [26,27]. Irrespective of the biological source, this large protein encompasses multiple distinct functional protein domains involved in mediating DSB, which includes three RuvC nuclease domains (RuvC-I, RuvC-II, and RuvC-III), three helical domains (Hel-I, Hel-II, and Hel-III), two L domains (L-I and L-II), one HNH nuclease domain, one arginine-rich bridge helix (Arg), and one C-terminal domain (CTD) [28].

There are two primary techniques for integrating CRISPR/Cas9 gene editing systems into the target genome of cells. The first method involves co-incubating Cas9 protein with sgRNA (or hybridized crRNA and tracrRNA) to form a complex known as Cas9–sgRNA (or Cas9–crRNA–tracrRNA). This complex is then introduced into the desired cells through microinjection [29]. Alternatively, the second approach utilizes the transfection of plasmids carrying genes that encode Cas9 proteins and produce sgRNA within the cells of interest using genetic engineering techniques. This enables the in vivo formation of a gene editing complex [30]. Once the integration step is accomplished, the Cas9–RNA complex functions like a cruise missile, searching for homologous target dsDNA sequences within in the genome. It selectively hybridizes with the complementary ssDNA of the target dsDNA, while the non-complementary ssDNA is displaced, ultimately forming an R-loop structure [31]. Subsequently, the Cas9 protein undergoes rapid conformational changes, leading to site-specific hydrolysis reactions on the phosphate backbone of the complementary ssDNA and the non-complementary ssDNA dependent on the HNH nuclease domain and RuvC nuclease domains, respectively [28]. Ultimately, this process results in the highly specific creation of a DSB at the target dsDNA within the genome, mediated by the CRISPR/Cas9 system.

### 3.2. DSB Repair and the Two Major Mechanisms

Although the aforementioned biochemical reactions eventually lead to DSB, they do not inherently enable gene editing without an essential additional step DNA repair. Perhaps, due to the excitement surrounding the establishment of the CRISPR/Cas system, the DNA repair process associated with DSBs and its impact on altering gene sequences can be easily overlooked in this field. However, DNA repair is a crucial process in various biological phenomena such as cancer, neurodegenerative diseases, and aging [32,33].

In general, both external factors (e.g., nucleic radiation) and internal factors (DNA metabolism during cell replication and differentiation) have a certain probability of causing DNA damage and breakage within cells, subsequently activating the cellular DNA repair mechanisms [32,34]. Given the abundance of DNA damage, there exists a diverse array of DNA repair pathways involving multiple and complex interactions between proteins and DNA [35]. The two primary modes of DSB repair currently known are on-homologous end joining (NHEJ) and homologous recombination (HR). NHEJ involves the ligation of broken DNA ends facilitated by the Ku70/80 protein [36]. On the other hand, HR utilizes a homologous dsDNA molecule as a template for repair [37]. In HR, the DNA ends undergoing DSB first undergo two 3′ end resections mediated by specific enzymes. Next, RecA/Rad51 family proteins facilitate the pairing of these two resected ends with homologous DNA regions within the genome, leading to the formation of two D-loops. Subsequently, these two D-loops extend their end resections through complementary ssDNA using DNA polymerase and are ligated to the phosphate group at the 5′ end of the opposing resected end by DNA ligase, resulting in the formation of a holiday junction (HJ). Finally, a series of DNA endonuclease and ligases resolve the HJ’s topology, separating it into two distinct dsDNA molecules to complete HR repair [38].

During the NHEJ pathway, exonucleases such as MRE11 are involved before the ligation process, resulting in the repaired DNA being slightly shorter compared to its pre-DSB state [39]. Additionally, the sequence of repaired dsDNA mediated by HR often differs from the original sequence primarily due to the resolution of HJ structure [40]. Consequently, the ultimate outcome of CRISPR/Cas9-mediated gene editing is the alteration of the DNA sequence through the DNA repair process.

## 4. Evolutions of the CRISPR/Cas System

In recent years, substantial advancements have been made in the field of genetic engineering, metagenomics, and protein purification technologies. These advancements have greatly contributed to progressive evolution of the CRISPR/Cas system. Current research endeavors primarily concentrate on the construction, exploration, and functional characterization of novel Cas proteins. The primary objective behind these efforts is to improve the efficacy and versatility of the CRISPR/Cas system for gene editing applications while also exploring its potential utilization in non-gene editing domains.

### 4.1. CRISPR/dCas-Induced Gene Silencing

Gene silencing mediated by dead Cas9 (dCas9) represents an early breakthrough in the evolution of the CRISPR/Cas system. It has gained widespread application in molecular biology laboratories for the precise regulation of protein expression levels in target genes within cells [41]. Small interfering RNA (siRNA)-based techniques have emerged as nucleic acid drugs for clinical treatments, addressing conditions such as polyneuropathies and atherosclerotic cardiovascular disease (ASCVD) [42,43]. However, siRNA-induced gene silencing operates at the transcriptome level rather than directly targeting the genome. Moreover, the potential off-target effects (OTEs) resulting from siRNA’s binding to non-target genes may lead to genotoxicity toward the intended therapeutic target [44]. Overcoming these limitations, dCas9-mediated gene silencing offers substantial advantages. Unlike Cas9 protein, dCas9 lacks functional HNH nuclease and RuvC nuclease domains, thereby preventing DSB upon complex formation between dCas9, sgRNA, and the target dsDNA [45]. Exploiting this property, anchoring the dCas9–sgRNA complex to a specific genomic position containing the target gene’s transcription start site (TSS), along with the necessary PAM sequence, enables effective gene silencing by impeding the activity of transcription factor proteins responsible for transcribing downstream genes into RNA. With this high specificity and affinity for dsDNA rather than RNA, the CRISPR/dCas9 system exhibits lower OTEs compared to siRNA-based gene silencing [46].

### 4.2. CRISPR/Cas-Mediated Point Mutation Gene Editing

Point Mutation, also referred to as single-base gene mutations, plays a significant role in the development of various anemias such as β-thalassemia, sickle cell anemia [47,48]. Additionally, point mutations in tumor suppressor proteins have been identified as causative factors in cancer formation [49]. Precise single-base gene editing holds great potential for treating diseases caused by these specific point mutations at the genetic level. Addressing this challenge, Komor et al. devised a strategy involving fusion protein consisting of dCas9 tethered to cytidine deaminase, enabling targeted single-base editing at specific genomic loci (C → T or G → A) [50]. The approach utilizes the fundamental principles of the CRISPR/dCas9, wherein fusion protein is positioned precisely at the target single base site within the genome. Subsequently, the tethered cytidine deaminase identifies and oxidize a site-specific cytosine, converting it to uracil (U). During subsequent DNA replication or repair processes, this U-carrying point mutation is transformed into a T, resulting in a complementary base change from G to A. Furthermore, the research group successfully integrated dCas9 with a hypothetical deoxyadenosine deaminase, enabling single-base editing of T → C or A → G in genomic DNA [51]. Notably, this CRISPR/dCas9-mediated single-base editing technique avoids causing DSB, thus minimizing additional burden on the gene editing process. Moreover, the concept of fusing Cas protein with other functional proteins for biological purposes has demonstrated its significance in various areas of research, including enhancing genome editing efficiency, improving homology-directed repair efficiency, live-cell imaging, prime editing, and epigenetic gene silencing [52,53,54,55,56].

### 4.3. Smaller CRISPR/Cas System Designation to Facilitate Gene Editing

As discussed earlier, the Cas9 protein has a substantial molecular weight of approximately 160 kDa, posing challenges for efficient integration of Cas9–sgRNA complexes or plasmids expressing the CRISPR/Cas9 system into cells [57]. To address this limitation, alternative Cas proteins with comparable functionality but smaller sizes have been identified successively. Notably, four distinct Cas proteins from different biological sources have emerged as mini CRISPR/Cas gene editing systems due to their reduced dimensions. These include Cpf1 (also known as Cas12a) derived from *Acidaminococcus* and *Lachnospiraceae* (~150 kDa) [58], Cas14 from uncultivated archaea (40–70 kDa) [59], CasΦ from huge phages (~70 kDa) [60], and Cas12f from *Acidibacillus sulfuroxidans* (approximately 61.5 kDa) [61]. Despite their smaller sizes, these Cas proteins retain similar functionality as Cas9 and exhibit robust target recognition capabilities when engaging with the target dsDNA during gene editing processes. The growing trend of identifying mini CRISPR/Cas systems suggests that further exploration will likely yield additional small Cas proteins, thereby facilitating more accessible and refined gene editing techniques.

## 5. Applications of the CRISPR/Cas System in Diseases Therapy and Diagnosis

The central dogma originally postulated that the functional characteristics of a protein are primarily determined its gene sequence. Exploiting this principle, the portable and robust CRISPR/Cas-mediated gene editing system, in conjunction with cellular immunobiology, has been extensively investigated and applied in immunotherapy. By modifying the functionality of key proteins involved, this approach aims to enhance therapeutic outcomes. Moreover, the development of CRISPR/Cas-based treatments directly targeting viral nucleic acids is gradually gaining traction. Recent advancements have unveiled novel Cas proteins that exhibit distinct nucleic acid interaction properties compared to Cas9. Apart from their gene editing applications, these newly discovered CRISPR/Cas systems have also been harnessed as elegant bioanalytical methods for highly sensitive disease biomarker detection.

### 5.1. Enhancement of New CAR-T Therapy

CAR-T therapy, which stands for chimeric antigen receptor T-cell therapy, is a cancer immunotherapy approach that enhances the immune response of specific T cells [62]. Essentially, CAR-T therapy involves genetically modifying T cells to express a chimeric antigen receptor, empowering them to more effectively target and eliminate cancer cells in patients [63]. However, current methods of gene fragment integration used in CAR-T therapy, such as γ-retroviral, can lead to abnormal functional phenotypes in modified T cells due to the random nature of gene insertion sites [64]. Thus, achieving precise gene insertion into the T-cell genome holds promise for optimizing CAR-T therapy efficacy. Leveraging the precise target recognition capabilities of the CRISPR/Cas9 system, analogous to a guided missile as previously mentioned (Section 3.1), Eyquem et al. successfully directed the CAR gene to integrate into the T-cell receptor α constant (TRAC) locus of T cells using CRISPR/Cas9, resulting in enhanced potency of CAR-T cells compared to previous methods [65]. Recently, Foy et al. developed a method utilizing CRISPR/Cas9 for precise gene insertion and multi-locus gene knockdown in T cells [66], demonstrating its clinical efficacy and safety. Moreover, for the treatment of T-cell acute lymphoblastic leukemia (T-ALL), single-base editing involving quadruple CRISPR/Cas gene editing of CAR-T has been developed, with related clinical trials receiving approved from the U.S. Food and Drug Administration (FDA) [67].

### 5.2. Establishment of Gene Editing-Drived Anti-HIV Viral Therapy

Acquired immunodeficiency syndrome (AIDS) is a disease caused by human immunodeficiency virus (HIV) infection, which leads to the destruction of immune cells in the body. HIV utilizes RNA as a template to efficiently carry out processes such as, reverse transcription, DNA integration, DNA replication, protein expression, and assembly of new viral particles within host cell [68]. Currently, the most effective treatment approach for AIDS involves a combination of multiple drugs, commonly known as cocktail therapy. This strategy simultaneously targets essential proteins involved in HIV replication using two or more drugs [69]. However, HIV exhibits high mutability, and the emergence of drug-resistant strains can lead to treatment failure due to the loss of inhibitory effects on target viral proteins within the cocktail therapy regimen. Furthermore, the development of chemical drugs typically entails a lengthy process of approximately ten to fifteen years, potentially resulting in delays in implementing cocktail therapy for AIDS [70].

In an innovative approach, Kaminski et al. focused on targeting the reverse transcribed DNA of HIV as a therapeutic strategy [71]. They employed the CRISPR/Cas system as a molecular tool to cleave HIV DNA and inhibit viral replication in vivo. The study involved designing a plasmid expressing saCas9, a smaller Cas protein compared to Cas9, along with multiple sgRNAs targeting HIV DNA. These components were delivered via an adeno-associated virus (AAV) delivery system, which was directed injected into HIV-1 Tg26 transgenic mice and rats. As a result, gene editing-mediated excision of HIV-1 DNA was achieved, leading to suppression of HIV RNAs levels in various tissues of disposed animals. Encouragingly, EBT-101, a CRISPR-based anti-HIV therapy, has obtained FDA approval for human clinical trials in AIDS treatment, highlighting the promising potential of the CRISPR/Cas system as an effective anti-HIV/AIDS therapeutic approach [72].

### 5.3. Development of New Biomarker Assays Based on New CRISPR/Cas Systems

The widespread application of the CRISPR/Cas system for biomarker analysis can be attributed to the discovery of two new Cas proteins, namely Cas13a and Cas12a. Cas13a, also known as C2c2, is derived from *Leptotrichia shahii* [73]. Unlike Cas9 which cleaves dsDNA, Cas13a has the unique ability to cleave single-stranded RNA (ssRNA) independent of the RNA sequence. This is accomplished through its RNA endonuclease domain, which is activated upon binding to crRNA and recognizing the target RNA [73]. The distinct properties of Cas13a have made it a powerful tool in nucleic acid biomarker detection, as demonstrated by Gootenberg et al.’s technology called specific high-sensitivity enzymatic reporter unlocking (SHERLOCK) [74]. In SHERLOCK, the target DNA (or RNA) undergoes initial amplification using a thermostatic exponential amplification method known as recombinase polymerase amplification (RPA) (for RNA targets, reverse transcription is performed prior to amplification). Subsequently, the amplified dsDNA is transcribed into ssRNA, which activates Cas13a through one T7 transcription step. Finally, the activated Cas13a cleaves the RNA probe containing fluorescent-quench groups, leading to the release of fluorescence. The magnitude of the fluorescence signal represents the copy number of the detected nucleic acid. SHERLOCK has been further enhanced and upgraded to version 2.0, enabling visual detection of Dengue and Zika virus ssRNA on detection strips with exceptional sensitivity (down to 2.0 aM) [75]. Notably, an FDA-authorized SARS-CoV-2 testing kit based on SHERLOCK 2.0 has been developed for viral RNA detection [76]. Cas13a has also found broad utility in the detection of other biomarker or virus, including microRNA, DNA methylation, Ebola, influenza A (H1N1), and hepatitis B virus (HBV) [77,78,79,80,81,82].

The utilization of CRISPR/Cas12a-based biomarker assays has attracted significant research interest. Cas12a, previously known as Cpf1, was identified in *Acidaminococcus* sp. [83]. Similar to Cas9, Cas12a processes gene editing capabilities and recognizes target dsDNA containing a PAM with the assistance of crRNA [84]. Upon successful recognition, the nuclease domain of Cas12a is exposed, leading to non-specific cleavage of ssDNA. Although Cas12a shares similarities with Cas13a, the main difference lies in their respective nuclease domains. Chen et al. reported the single-stranded DNase activity of Cas12a and developed a strategy called DNA endonuclease targeted CRISPR trans Reporter (DETECTR) for the analysis of human papillomavirus (HPV) in patient samples [84]. In principle, DETECTR follows a similar experimental approach to is SHERLOCK, combining RPA with the CRISPR/Cas system to achieve highly sensitive nucleic acid detection. However, the use of DNA probes in DETECTR offers practical advantages in terms of experimental manipulation and clinical testing due to their higher thermal stability and cost-effectiveness compared to RNA probes. Consequently, recent bioanalytical investigations employing CRISPR/Cas12a have become more prevalent than those using CRISPR/Cas13a, with a focus on substituting nucleic acid amplification methods other than RPA and optimizing nucleic acid probes or detection techniques to meet requirements of highly sensitive analysis for various biomarkers [85,86,87,88,89].

## 6. Prospects

The discovery of new Cas proteins holds significant importance in the field of gene editing, gene therapy, and biomarker diagnostics. Over the past decade, nearly every discovered Cas protein has contributed to the emergence of a new CRISPR/Cas system that finds wide applications in these domains. Consequently, utilizing macrogenomics, molecular biology and cryogenic electron microscopy (Cryo-EM) techniques to identify novel Cas proteins or functionally analogous proteins has become a prominent research focus for subsequent investigations [90,91]. Another crucial area of concern is the occurrence of off-target effects during gene editing with existing CRISPR/Cas systems. Although some technical methods have been developed to mitigate these effects, there remains an urgent need to further reduce the genotoxicity associated with CRISPR/Cas applications [92]. Moreover, as CRISPR/Cas systems are poised to be developed into marketable drugs in the near future, it is imperative to conduct pharmacological and pharmacokinetic studies. The development of bioanalytical methods and corresponding guidelines will facilitate the assessment of efficacy, ADME (abbreviation of absorption, distribution, metabolism, and excretion) properties, and safety of CRISPR/Cas-associated drugs. Additionally, due to the substantial molecular weight of CRISPR/Cas systems, the design of safe and targeted drug delivery systems is paramount. Furthermore, it is essential not to overlook the social and ethical implications raised by the CRISPR/Cas system in recent years, particularly concerning its powerful gene editing capabilities. These issues often arise from decisions regarding gene editing targets such as human embryonic cells [93]. Consequently, while the clinical application of CRISPR/Cas shows promise, the development of a matching gene editing therapeutic monitoring system is urgently required.

## 7. Conclusions

In summary, this essay endeavors to provide a structured and readily understandable overview of the discovery history, molecular mechanisms, evolutionary developments, and clinical applications of the CRISPR/Cas system. Over time, the continuous advancement in understanding and application has led the CRISPR/Cas system to find extensive utilization in laboratory research, immunotherapy, antiviral therapy, agriculture, and other fields. While concerns regarding off-target effects and ethical considerations surrounding CRISPR/Cas gene editing systems haven arisen in recent years, the significant contributions of the CRISPR/Cas system to various domains appear to outweigh these challenges. Furthermore, with ongoing in-depth research and further utilization of the CRISPR/Cas system in the future, it is expected that related issues will be effectively addressed. It has been just over 12 and 4 years since the molecular mechanism of CRISPR/Cas9 was unveiled and its contributors, Jennifer Doudna and Emmanuelle Charpentier, were awarded the Nobel Prize in Chemistry, respectively. The tremendous potential of the CRISPR/Cas macromolecular system in biomedical research heralds an exciting decade of even greater advancements and achievements, and this review hopes to lay a foundation for understanding and embracing these progresses.

## Data Availability

The raw data supporting the conclusions of this article will be made available by the authors on request.

## References

[1] Crick F. (1970). Central Dogma of Molecular Biology. Nature.

[2] Cobb M., Comfort N. (2023). What Rosalind Franklin Truly Contributed to the Discovery of DNA’s Structure. Nature.

[3] Gibbs R.A. (2020). The Human Genome Project Changed Everything. Nat. Rev. Genet..

[4] Beck S. (2014). The Human Epigenome Project: Past, Present, and Future. Reference Module in Biomedical Sciences.

[5] Yin W., Mao C., Luan X., Shen D.-D., Shen Q., Su H., Wang X., Zhou F., Zhao W., Gao M. (2020). Structural Basis for Inhibition of the RNA-Dependent RNA Polymerase from SARS-CoV-2 by Remdesivir. Science.

[6] Malech H.L., Notarangelo L.D. (2023). Gene Therapy for Inborn Errors of Immunity: Base Editing Comes into Play. Cell.

[7] Jang G., Shin H.R., Do H.-S., Kweon J., Hwang S., Kim S., Heo S.H., Kim Y., Lee B.H. (2023). Therapeutic Gene Correction for Lesch-Nyhan Syndrome Using CRISPR-Mediated Base and Prime Editing. Mol. Ther. Nucleic Acids.

[8] Uranga M., Aragonés V., Daròs J.-A., Pasin F. (2023). Heritable CRISPR-Cas9 Editing of Plant Genomes Using RNA Virus Vectors. STAR Protoc..

[9] Jin D.-Y., Chen X., Liu Y., Williams C.M., Pedersen L.C., Stafford D.W., Tie J.-K. (2023). A Genome-Wide CRISPR-Cas9 Knockout Screen Identifies FSP1 as the Warfarin-Resistant Vitamin K Reductase. Nat. Commun..

[10] Fudge J.B. (2023). Cardiac Defect Corrected in Vivo with CRISPR. Nat. Biotechnol..

[11] Tang Y., Gao L., Feng W., Guo C., Yang Q., Li F., Le X.C. (2021). The CRISPR–Cas Toolbox for Analytical and Diagnostic Assay Development. Chem. Soc. Rev..

[12] Ishino Y., Shinagawa H., Makino K., Amemura M., Nakata A. (1987). Nucleotide Sequence of the Iap Gene, Responsible for Alkaline Phosphatase Isozyme Conversion in Escherichia Coli, and Identification of the Gene Product. J. Bacteriol..

[13] Jansen R., van Embden J.D.A., Gaastra W., Schouls L.M. (2002). Identification of Genes That Are Associated with DNA Repeats in Prokaryotes. Mol. Microbiol..

[14] Lillestøl R.K., Redder P., Garrett R.A., Brügger K. (2006). A Putative Viral Defence Mechanism in Archaeal Cells. Archaea.

[15] Mojica F.J., Díez-Villaseñor C.S., García-Martínez J., Soria E. (2005). Intervening Sequences of Regularly Spaced Prokaryotic Repeats Derive from Foreign Genetic Elements. J. Mol. Evol..

[16] Haft D.H., Selengut J., Mongodin E.F., Nelson K.E. (2005). A Guild of 45 CRISPR-Associated (Cas) Protein Families and Multiple CRISPR/Cas Subtypes Exist in Prokaryotic Genomes. PLoS Comput. Biol..

[17] Marraffini L.A., Sontheimer E.J. (2010). CRISPR Interference: RNA-Directed Adaptive Immunity in Bacteria and Archaea. Nat. Rev. Genet..

[18] Marraffini L.A., Sontheimer E.J. (2010). Self versus Non-Self Discrimination during CRISPR RNA-Directed Immunity. Nature.

[19] Garneau J.E., Dupuis M.-È., Villion M., Romero D.A., Barrangou R., Boyaval P., Fremaux C., Horvath P., Magadán A.H., Moineau S. (2010). The CRISPR/Cas Bacterial Immune System Cleaves Bacteriophage and Plasmid DNA. Nature.

[20] Deltcheva E., Chylinski K., Sharma C.M., Gonzales K., Chao Y., Pirzada Z.A., Eckert M.R., Vogel J., Charpentier E. (2011). CRISPR RNA Maturation by Trans-Encoded Small RNA and Host Factor RNase III. Nature.

[21] Jinek M., Chylinski K., Fonfara I., Hauer M., Doudna J.A., Charpentier E. (2012). A Programmable Dual-RNA–Guided DNA Endonuclease in Adaptive Bacterial Immunity. Science.

[22] Cong L., Ran F.A., Cox D., Lin S., Barretto R., Habib N., Hsu P.D., Wu X., Jiang W., Marraffini L.A. (2013). Multiplex Genome Engineering Using CRISPR/Cas Systems. Science.

[23] Bin Moon S., Lee J.M., Kang J.G., Lee N.-E., Ha D.-I., Kim D.Y., Kim S.H., Yoo K., Kim D., Ko J.-H. (2018). Highly Efficient Genome Editing by CRISPR-Cpf1 Using CRISPR RNA with a Uridinylate-Rich 3′-Overhang. Nat. Commun..

[24] Chylinski K., Le Rhun A., Charpentier E. (2013). The TracrRNA and Cas9 Families of Type II CRISPR-Cas Immunity Systems. RNA Biol..

[25] Liao C., Beisel C.L. (2021). The TracrRNA in CRISPR Biology and Technologies. Annu. Rev. Genet..

[26] Jinek M., Jiang F., Taylor D.W., Sternberg S.H., Kaya E., Ma E., Anders C., Hauer M., Zhou K., Lin S. (2014). Structures of Cas9 Endonucleases Reveal RNA-Mediated Conformational Activation. Science.

[27] Dooley S.K., Baken E.K., Moss W.N., Howe A., Young J.K. (2021). Identification and Evolution of Cas9 TracrRNAs. CRISPR J..

[28] Jiang F., Doudna J.A. (2017). CRISPR–Cas9 Structures and Mechanisms. Annu. Rev. Biophys..

[29] Iyer J., DeVaul N., Hansen T., Nebenfuehr B. (2019). Using Microinjection to Generate Genetically Modified Caenorhabditis Elegans by CRISPR/Cas9 Editing. Microinjection.

[30] Kolasinliler G., Aagre M.M., Akkale C., Kaya H.B. (2023). The Use of CRISPR-Cas-Based Systems in Bacterial Cell Factories. Biochem. Eng. J..

[31] Handelmann C.R., Tsompana M., Samudrala R., Buck M.J. (2023). The Impact of Nucleosome Structure on CRISPR/Cas9 Fidelity. Nucleic Acids Res..

[32] Hoeijmakers J.H.J. (2009). DNA Damage, Aging, and Cancer. N. Engl. J. Med..

[33] Rass U., Ahel I., West S.C. (2007). Defective DNA Repair and Neurodegenerative Disease. Cell.

[34] Chatterjee N., Walker G.C. (2017). Mechanisms of DNA Damage, Repair and Mutagenesis. Environ. Mol. Mutagen..

[35] Bader A.S., Hawley B.R., Wilczynska A., Bushell M. (2020). The Roles of RNA in DNA Double-Strand Break Repair. Br. J. Cancer.

[36] Zhao B., Watanabe G., Morten M.J., Reid D.A., Rothenberg E., Lieber M.R. (2019). The Essential Elements for the Noncovalent Association of Two DNA Ends during NHEJ Synapsis. Nat. Commun..

[37] San Filippo J., Sung P., Klein H. (2008). Mechanism of Eukaryotic Homologous Recombination. Annu. Rev. Biochem..

[38] Wright W.D., Shah S.S., Heyer W.-D. (2018). Homologous Recombination and the Repair of DNA Double-Strand Breaks. J. Biol. Chem..

[39] Rotheneder M., Stakyte K., van de Logt E., Bartho J.D., Lammens K., Fan Y., Alt A., Kessler B., Jung C., Roos W.P. (2023). Cryo-EM Structure of the Mre11-Rad50-Nbs1 Complex Reveals the Molecular Mechanism of Scaffolding Functions. Mol. Cell.

[40] Shah Punatar R., Martin M.J., Wyatt H.D.M., Chan Y.W., West S.C. (2017). Resolution of Single and Double Holliday Junction Recombination Intermediates by GEN1. Proc. Natl. Acad. Sci. USA.

[41] El-Sappah A.H., Yan K., Huang Q., Islam M.M., Li Q., Wang Y., Khan M.S., Zhao X., Mir R.R., Li J. (2021). Comprehensive Mechanism of Gene Silencing and Its Role in Plant Growth and Development. Front. Plant Sci..

[42] Miname M.H., Rocha V.Z., Santos R.D. (2021). The Role of RNA-Targeted Therapeutics to Reduce ASCVD Risk: What Have We Learned Recently?. Curr. Atheroscler. Rep..

[43] Adams D., Gonzalez-Duarte A., O’Riordan W.D., Yang C.-C., Ueda M., Kristen A.V., Tournev I., Schmidt H.H., Coelho T., Berk J.L. (2018). Patisiran, an RNAi Therapeutic, for Hereditary Transthyretin Amyloidosis. N. Engl. J. Med..

[44] Fedorov Y., Anderson E.M., Birmingham A., Reynolds A., Karpilow J., Robinson K., Leake D., Marshall W.S., Khvorova A. (2006). Off-Target Effects by SiRNA Can Induce Toxic Phenotype. RNA.

[45] Wang C., Qu Y., Cheng J.K.W., Hughes N.W., Zhang Q., Wang M., Cong L. (2022). DCas9-Based Gene Editing for Cleavage-Free Genomic Knock-in of Long Sequences. Nat. Cell Biol..

[46] Boettcher M., McManus M.T. (2015). Choosing the Right Tool for the Job: RNAi, TALEN or CRISPR. Mol. Cell.

[47] Zulkeflee R.H., Bahar R., Abdullah M., Mohd Radzi M.A.R., Md Fauzi A., Hassan R. (2023). Application of Targeted Next-Generation Sequencing for the Investigation of Thalassemia in a Developing Country: A Single Center Experience. Diagnostics.

[48] Bishop M.F., Ferrone F.A. (2023). The Sickle-Cell Fiber Revisited. Biomolecules.

[49] Frankell A.M., Dietzen M., Al Bakir M., Lim E.L., Karasaki T., Ward S., Veeriah S., Colliver E., Huebner A., Bunkum A. (2023). The Evolution of Lung Cancer and Impact of Subclonal Selection in TRACERx. Nature.

[50] Komor A.C., Kim Y.B., Packer M.S., Zuris J.A., Liu D.R. (2016). Programmable Editing of a Target Base in Genomic DNA without Double-Stranded DNA Cleavage. Nature.

[51] Gaudelli N.M., Komor A.C., Rees H.A., Packer M.S., Badran A.H., Bryson D.I., Liu D.R. (2017). Programmable Base Editing of A•T to G•C in Genomic DNA without DNA Cleavage. Nature.

[52] Park J., Yoon J., Kwon D., Han M.-J., Choi S., Park S., Lee J., Lee K., Lee J., Lee S. (2021). Enhanced Genome Editing Efficiency of CRISPR PLUS: Cas9 Chimeric Fusion Proteins. Sci. Rep..

[53] Li G., Wang H., Zhang X., Wu Z., Yang H. (2021). A Cas9-Transcription Factor Fusion Protein Enhances Homology-Directed Repair Efficiency. J. Biol. Chem..

[54] Ye H., Jiang C., Li L., Li H., Rong Z., Lin Y. (2022). Live-Cell Imaging of Genomic Loci with Cas9 Variants. Biotechnol. J..

[55] Zhao Z., Shang P., Mohanraju P., Geijsen N. (2023). Prime Editing: Advances and Therapeutic Applications. Trends Biotechnol..

[56] Nuñez J.K., Chen J., Pommier G.C., Cogan J.Z., Replogle J.M., Adriaens C., Ramadoss G.N., Shi Q., Hung K.L., Samelson A.J. (2021). Genome-Wide Programmable Transcriptional Memory by CRISPR-Based Epigenome Editing. Cell.

[57] Glass Z., Lee M., Li Y., Xu Q. (2018). Engineering the Delivery System for CRISPR-Based Genome Editing. Trends Biotechnol..

[58] Zetsche B., Gootenberg J.S., Abudayyeh O.O., Slaymaker I.M., Makarova K.S., Essletzbichler P., Volz S.E., Joung J., van der Oost J., Regev A. (2015). Cpf1 Is a Single RNA-Guided Endonuclease of a Class 2 CRISPR-Cas System. Cell.

[59] Harrington L.B., Burstein D., Chen J.S., Paez-Espino D., Ma E., Witte I.P., Cofsky J.C., Kyrpides N.C., Banfield J.F., Doudna J.A. (2018). Programmed DNA Destruction by Miniature CRISPR-Cas14 Enzymes. Science.

[60] Pausch P., Al-Shayeb B., Bisom-Rapp E., Tsuchida C.A., Li Z., Cress B.F., Knott G.J., Jacobsen S.E., Banfield J.F., Doudna J.A. (2020). CRISPR-CasΦ from Huge Phages Is a Hypercompact Genome Editor. Science.

[61] Wu Z., Zhang Y., Yu H., Pan D., Wang Y., Wang Y., Li F., Liu C., Nan H., Chen W. (2021). Programmed Genome Editing by a Miniature CRISPR-Cas12f Nuclease. Nat. Chem. Biol..

[62] Sterner R.C., Sterner R.M. (2021). CAR-T Cell Therapy: Current Limitations and Potential Strategies. Blood Cancer J..

[63] Ye B., Stary C.M., Li X., Gao Q., Kang C., Xiong X. (2018). Engineering Chimeric Antigen Receptor-T Cells for Cancer Treatment. Mol. Cancer.

[64] Liu J., Zhou G., Zhang L., Zhao Q. (2019). Building Potent Chimeric Antigen Receptor T Cells with CRISPR Genome Editing. Front. Immunol..

[65] Eyquem J., Mansilla-Soto J., Giavridis T., van der Stegen S.J.C., Hamieh M., Cunanan K.M., Odak A., Gönen M., Sadelain M. (2017). Targeting a CAR to the TRAC Locus with CRISPR/Cas9 Enhances Tumour Rejection. Nature.

[66] Foy S.P., Jacoby K., Bota D.A., Hunter T., Pan Z., Stawiski E., Ma Y., Lu W., Peng S., Wang C.L. (2023). Non-Viral Precision T Cell Receptor Replacement for Personalized Cell Therapy. Nature.

[67] Salas-Mckee J., Kong W., Gladney W.L., Jadlowsky J.K., Plesa G., Davis M.M., Fraietta J.A. (2019). CRISPR/Cas9-Based Genome Editing in the Era of CAR T Cell Immunotherapy. Hum. Vaccines Immunother..

[68] Masuda T., Kotani O., Yokoyama M., Abe Y., Kawai G., Sato H. (2022). Cis-Allosteric Regulation of HIV-1 Reverse Transcriptase by Integrase. Viruses.

[69] Maenza J., Flexner C. (1998). Combination Antiretroviral Therapy for HIV Infection. Am. Fam. Physician.

[70] Lavecchia A. (2019). Deep Learning in Drug Discovery: Opportunities, Challenges and Future Prospects. Drug Discov. Today.

[71] Kaminski R., Bella R., Yin C., Otte J., Ferrante P., Gendelman H.E., Li H., Booze R., Gordon J., Hu W. (2016). Excision of HIV-1 DNA by Gene Editing: A Proof-of-Concept in Vivo Study. Gene Ther..

[72] Allen A.G., Chung C.-H., Worrell S.D., Nwaozo G., Madrid R., Mele A.R., Dampier W., Nonnemacher M.R., Wigdahl B. (2023). Assessment of Anti-HIV-1 Guide RNA Efficacy in Cells Containing the Viral Target Sequence, Corresponding GRNA, and CRISPR/Cas9. Front. Genome Ed..

[73] Liu L., Li X., Wang J., Wang M., Chen P., Yin M., Li J., Sheng G., Wang Y. (2017). Two Distant Catalytic Sites Are Responsible for C2c2 RNase Activities. Cell.

[74] Gootenberg J.S., Abudayyeh O.O., Lee J.W., Essletzbichler P., Dy A.J., Joung J., Verdine V., Donghia N., Daringer N.M., Freije C.A. (2017). Nucleic Acid Detection with CRISPR-Cas13a/C2c2. Science.

[75] Gootenberg J.S., Abudayyeh O.O., Kellner M.J., Joung J., Collins J.J., Zhang F. (2018). Multiplexed and Portable Nucleic Acid Detection Platform with Cas13, Cas12a, and Csm6. Science.

[76] Biosciences S. (2020). Sherlock^TM^ CRISPR SARS-CoV-2 Kit.

[77] Zhou H., Bu S., Xu Y., Xue L., Li Z., Hao Z., Wan J., Tang F. (2022). CRISPR/Cas13a Combined with Hybridization Chain Reaction for Visual Detection of Influenza A (H1N1) Virus. Anal. Bioanal. Chem..

[78] Ding R., Shen Y., Yuan M., Zheng X., Chen S., Duan G. (2022). Rapid and Facile Detection of HBV with CRISPR/Cas13a. New J. Chem..

[79] Zhou T., Huang M., Lin J., Huang R., Xing D. (2021). High-Fidelity CRISPR/Cas13a Trans-Cleavage-Triggered Rolling Circle Amplified DNAzyme for Visual Profiling of MicroRNA. Anal. Chem..

[80] Wang X., Zhou S., Chu C., Yang M., Huo D., Hou C. (2021). Dual Methylation-Sensitive Restriction Endonucleases Coupling with an RPA-Assisted CRISPR/Cas13a System (DESCS) for Highly Sensitive Analysis of DNA Methylation and Its Application for Point-of-Care Detection. ACS Sens..

[81] Barnes K.G., Lachenauer A.E., Nitido A., Siddiqui S., Gross R., Beitzel B., Siddle K.J., Freije C.A., Dighero-Kemp B., Mehta S.B. (2020). Deployable CRISPR-Cas13a Diagnostic Tools to Detect and Report Ebola and Lassa Virus Cases in Real-Time. Nat. Commun..

[82] Liu X., Kang X., Lei C., Ren W., Liu C. (2022). Programming the Trans-Cleavage Activity of CRISPR-Cas13a by Single-Strand DNA Blocker and Its Biosensing Application. Anal. Chem..

[83] Makarova K.S., Wolf Y.I., Alkhnbashi O.S., Costa F., Shah S.A., Saunders S.J., Barrangou R., Brouns S.J.J., Charpentier E., Haft D.H. (2015). An Updated Evolutionary Classification of CRISPR–Cas Systems. Nat. Rev. Microbiol..

[84] Chen J.S., Ma E., Harrington L.B., Da Costa M., Tian X., Palefsky J.M., Doudna J.A. (2018). CRISPR-Cas12a Target Binding Unleashes Indiscriminate Single-Stranded DNase Activity. Science.

[85] Long W., Yang J., Zhao Q., Pan Y., Luan X., He B., Han X., Wang Y., Song Y. (2023). Metal–Organic Framework-DNA Bio-Barcodes Amplified CRISPR/Cas12a Assay for Ultrasensitive Detection of Protein Biomarkers. Anal. Chem..

[86] Gong S., Wang X., Zhou P., Pan W., Li N., Tang B. (2022). AND Logic-Gate-Based CRISPR/Cas12a Biosensing Platform for the Sensitive Colorimetric Detection of Dual MiRNAs. Anal. Chem..

[87] Chen Y., Xu X., Wang J., Zhang Y., Zeng W., Liu Y., Zhang X. (2022). Photoactivatable CRISPR/Cas12a Strategy for One-Pot DETECTR Molecular Diagnosis. Anal. Chem..

[88] Zhang W., Shi R., Dong K., Hu H., Shu W., Mu Y., Yan B., Li L., Xiao X., Wang H. (2022). The Off-Target Effect of CRISPR-Cas12a System toward Insertions and Deletions between Target DNA and CrRNA Sequences. Anal. Chem..

[89] Zeng R., Gong H., Li Y., Li Y., Lin W., Tang D., Knopp D. (2022). CRISPR-Cas12a-Derived Photoelectrochemical Biosensor for Point-Of-Care Diagnosis of Nucleic Acid. Anal. Chem..

[90] Alkhnbashi O.S., Mitrofanov A., Bonidia R., Raden M., Tran V.D., Eggenhofer F., Shah S.A., Öztürk E., Padilha V.A., Sanches D.S. (2021). CRISPRloci: Comprehensive and Accurate Annotation of CRISPR–Cas Systems. Nucleic Acids Res..

[91] Saito M., Xu P., Faure G., Maguire S., Kannan S., Altae-Tran H., Vo S., Desimone A., Macrae R.K., Zhang F. (2023). Fanzor Is a Eukaryotic Programmable RNA-Guided Endonuclease. Nature.

[92] Naeem M., Majeed S., Hoque M.Z., Ahmad I. (2020). Latest Developed Strategies to Minimize the Off-Target Effects in CRISPR-Cas-Mediated Genome Editing. Cells.

[93] Ledford H. (2023). Why CRISPR Babies Are Still Too Risky—Embryo Studies Highlight Challenges. Nature.

